# New Highly Sulfonated Polythioethers as Polyelectrolyte Membranes for Water Electrolysis

**DOI:** 10.1021/acspolymersau.4c00079

**Published:** 2025-01-23

**Authors:** Ignasi de Azpiazu Nadal, Bruno Branco, Günter E.M. Tovar, Jochen Kerres, René A. J. Janssen, Stéphanie Reynaud, Vladimir Atanasov

**Affiliations:** a Institute of Chemical Process Engineering ICVT, University of Stuttgart, Boeblinger Str. 78, Stuttgart 70199, Germany; b IPREM, UMR5254, CNRS/Universite de Pau et des Pays de l’Adour, E2S UPPA, Pau 64053, France; c Molecular Materials and Nanosystems, Institute of Complex Molecular Systems, Eindhoven University of Technology, P.O. Box 513, Eindhoven 5600 MB, The Netherlands; d Institute of Interfacial Process Engineering and Plasma Technology IGVP, University of Stuttgart, Pfaffenwaldring 31, Stuttgart 70569, Germany; e Fraunhofer Institute for Interfacial Engineering and Biotechnology IGB, Nobelstr. 12, Stuttgart 70569, Germany; f Forschungszentrum Jülich GmbH, Helmholtz Institute Erlangen-Nürnberg for Renewable Energy (IEK-11), Cauerstr. 1, Erlangen 91058, Germany; g Chemical Resource Beneficiation, Faculty of Natural Sciences, North-West University, Potchefstroom 2520, South Africa; h Dutch Institute for Fundamental Energy Research, De Zaale 20, Eindhoven 5612 AJ, The Netherlands

**Keywords:** fluorinated poly(arylene thioether), proton-exchange membranes, sulfonated ionomers, polycondensation, water electrolysis, catalyst optimization

## Abstract

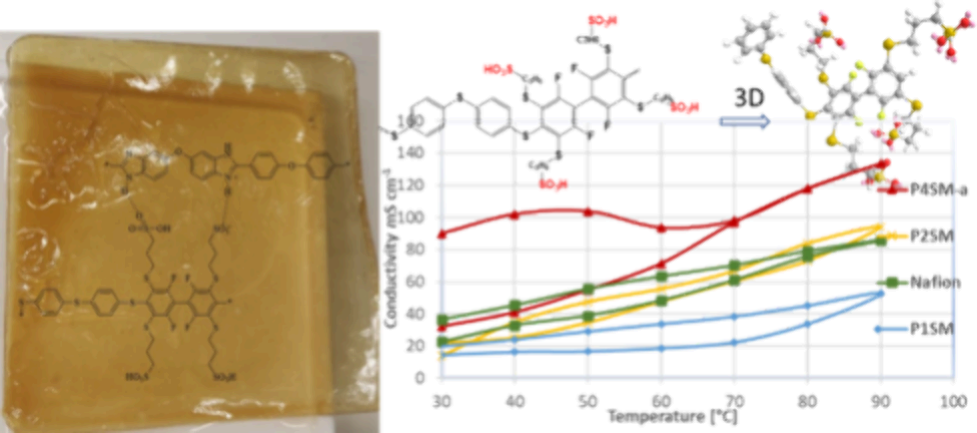

Herein, the synthesis and characterization of highly sulfonated poly(arylene thioethers) for application as polymer electrolyte membranes in water electrolysis are reported. In a first step, poly(arylene thioethers) were obtained by using mild reaction conditions of a polycondensation reaction between 4,4′-thiobisbenzenethiol and decafluorobiphenyl. In a second step, the resulting poly(arylene thioethers) were sulfonated by a fluorothiol displacement click reaction of the fluorinated monomers by sodium 3-mercapto-1-propanesulfonate. Thus, highly sulfonated polymers were obtained, resulting in water-soluble ionomers. Stable polymer electrolyte membranes with enhanced thermal and chemical stability were attained by blending ionomers with a poly(benzimidazole) derivative (PBI-OO). The resulting proton-exchange membranes (PEMs) based on the new sulfonated ionomer PBI-OO blends showed about 40% higher proton conductivity than Nafion at 90 °C. The proton-conducting membranes with the highest conductivity and best film-forming properties were applied for water electrolysis. Combined with optimized water oxidation and reduction catalysts, the selected tetra-sulfonated polymer-based PEM reached 1.784 V at 1 A cm^–2^ in the electrolysis of pure water.

## Introduction

Proton-exchange membranes (PEMs) play a key role in electrode separation and proton transport in electrolytic cells. The number of publications dealing with PEMs for water electrolysis has increased exponentially and reached about 2500 in 2021.^1^ PEMs typically comprise polymers with a hydrophobic backbone, carrying acidic functional groups that can conduct and deliver protons. The most popular and commercially available PEMs are currently Nafion from Dupont and Aquivion from Solvay. Both consist of perfluorinated backbones with sulfonic acid groups at the end of a perfluoroalkyl side chain exhibiting high chemical resistance and high proton conductivity (0.1–0.2 S cm^–1^). Drawbacks are the high costs (>1000 $ m^–2^) and narrow operational window being in the range of 30–90 °C and 50–100% RH.^2^ Moreover, per- and polyfluoroalkyl substances (PFAS) to which the perfluorosulfonic acids (PFSAs) belong are currently discussed to be banned from production and use due to environmental and health concerns. Within this context, the research community has been developing innovative membranes operating at intermediate temperatures (80–120 °C). Focus has been laid on hydrocarbon-sulfonated aromatic polymers offering such advantages as H-bond, ion-counterion, and π-stacking interactions of aromatic moieties leading to good film-forming properties, with high thermal and chemical stability.^3^ Moreover, the incorporation of fluorine atoms in the polymer backbone has also been shown to improve the thermal stability via the high stability of the C–F bond.^4^ Simultaneously, the high electronegativity of fluorine stabilizes the dissociation of the sulfonic acid groups by increasing the electron withdrawing effect, leading to a low p*K*_a_ value of −5.5.^5,6^ The latter favors simultaneously the mobility and solubility of the permeants in the polymer matrix when incorporated into the amorphous polymer structures due to high free volume and low cohesion energy.^7^ Schuster et al.^8^ have taken the advantage of the aromatic backbone and have reported the use of arylene ionomers based on polysulfones to reach high thermooxidative and hydrolytic stability. Although polysulfones possess a more rigid structure than poly(thioethers), the latter have shown high chemical and heat resistance. Katzfuss et al.^9^ have synthesized a partially fluorinated and sulfonated poly(arylene sulfone), which, when being ionically cross-linked with polybenzimidazole (PBI), has shown good proton conductivity. This work has followed the strategy of the Kerres group,^10^ using various monomers for the synthesis of new sulfonated polysulfide/sulfone polymers suitable for PEM fuel cells. Takamuku et al.^11^ have reported the synthesis of partially fluorinated poly(arylene thioether)s in mild base-mediated polycondensations of decafluorobiphenyl (DFBP) and 4,4′-thiobisbenzenethiol (TBBT). Poly(arylene thioether)s have been obtained from equimolar comonomer quantities with 1.5 equiv of potassium carbonate at 80 °C for 17 h. The molecular weights of the obtained polythioethers have not been reported, but a Mn of 5.7 kDa, a Mw of 12.1 kDa, and a dispersity *Đ* = 2.13 have been reported for the final polyelectrolytes obtained after an oxidation of the thioether and the subsequently introduced thiol functional groups. On the other hand, Park et al.^12^ have reported the synthesis of similar poly(arylene thioether)s. In this approach, trimethylsilyl (TMS)-protected thioethers synthesized prior to the polycondensation step have been used. Polymerization of DFBP and TMS-TBBT comonomers has been reported in DMF at room temperature (RT) for 5 min. This resulted in polymers having Mw = 47 kDa and high dispersity *Đ* = 6.05, which has been attributed to cross-linking or branching side reactions.

In this study, we were aiming to apply this knowledge and develop a more advanced sulfonation procedure targeting high degree sulfonations without decomposition of the polymer backbone. Therefore, in the first step, a polycondensation reaction of DFBP with TBBT was utilized to prepare the backbone. Synthesis parameters were optimized to reach high molar masses with control of the polymer dispersity (*Đ* ≤ 3). In the second step, we applied our innovative sulfonation method using sodium 3-mercapto-1-propanesulfonate at very mild conditions (RT, 48 h).^13^ A highly sulfonated polymer was targeted to favor proton channel formation, achieving up to four sulfonic acid groups per monomer unit. In the last step, ionic cross-linking of the sulfonated polymer with polybenzimidazole bearing two ether linkages (PBI-OO) was used to prepare stable proton-conductive membranes. The membranes showed good proton conductivity ranging from 64 to 202 mS cm^–1^ at room temperature. Finally, a blend membrane based on the tetra-sulfonated polymer was selected for water electrolysis application reaching 1.784 V at 1 A cm^–2^ for a water temperature of 60 °C.

## Experimental Section

### Materials

All materials were used as received unless noted. Dimethyl sulfoxide (DMSO), anhydrous quality *N*,*N*-dimethylacetamide (DMAc) (<0.005% H2O, 99.5%), 4,4′-thiosbisbenzenethiol (TBBT, 98%), decafluorobiphenyl (DFBP, 99%), potassium carbonate (K_2_CO_3_, ≥99%), sodium 3-mercapto-1-propanesulfonate (SMPS, 90%), and 1,8-diazabicyclo[5.4.0]undec-7-ene (DBU, 98%) were purchased from Sigma-Aldrich. PBI-OO (poly-[(1-(4,4′-diphenylether)-5-oxybenzimidazole)-benzimidazole]) was supplied by Fumatech. Nafion membrane NRE–212 was purchased from Ion Power. Pt/C (40 wt %) was purchased from Sigma-Aldrich. RuO_2_ (anhydrous, 99.9%) and Nafion dispersion (D-521) were purchased from Alfa Aesar. RuO_2_ was stored in an inert atmosphere. 2-Propanol was purchased from Biosolve. All catalyst inks were prepared using water purified in a Millipore system (ρ > 18 MΩ cm).

### Instrumentation

NMR spectra were recorded on a Bruker Advance 400 spectrometer at a resonance frequency of 400.1 MHz for ^1^H and 376.5 MHz for ^19^F NMR at RT. The polymer average molar masses (*M*_n_, *M*_w_) and dispersities (*Đ*) were determined by size-exclusion chromatography (SEC) using an Agilent Technology SEC system (Series 1200) coupled with a viscosity detector (PSS ETA-2010) and a refractive index detector (Shodex RI71). A set of three PSS GRAM columns (30, 3000, and 3000 Å) were used and calibrated with a series of polystyrene standards; DMAc containing 0.05 M LiBr has been used as an eluent. All of the samples were filtered by a Whatman syringe filter over a microporous PTFE membrane (0.45 μm, Whatman 6878-2510) before being injected into the column system. The thermal stability of the polymer membranes was determined by thermogravimetric analysis (TGA, Netzsch, model STA 449C) with a heating rate of 20 K min^–1^ for the range 20–600 °C under an atmosphere enriched with oxygen (65–70% O_2_, 35–30% N_2_). The TGA is connected to an FTIR (Bruker) spectrometer. The vapors from the TGA chamber are transferred to the FTIR analyzer, which is continuously scanning for any gaseous degradation products. Gram–Schmidt signal (total FTIR signal) and absorbance at 1360 cm^–1^, corresponding to SO3 vibration resonance, were recorded during the TGA measurements. Ion-exchange capacities (IEC_Titr._) were determined by a titration. Membranes in the H+ form were immersed in saturated sodium chloride solution (NaCl) for 24 h. The liberated ions were then titrated with a 0.1 M NaOH solution to an equivalent point (IEC_Titr._).
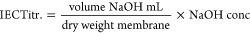


The specific resistance (*R*_spec_) of the membranes was determined at RT in 0.5 M HCl solution by electrochemical impedance spectroscopy (EIS) using a method described in the literature^14^ on an IM6Model of Zahner Elektrik. EIS measurements at different temperatures 30–90 °C under controlled RH were performed on a MTS740 Scribner test station. SEM was recorded using a HIROX SH-3000 scanning electron microscope, and samples were spattered in a sputtering chamber with Au at 30 mA for 60 s prior to use.

### Synthesis of PolyFAT

In a round-bottom reaction flask (500 mL) equipped with a reflux condenser and inert gas in/outlet and a mechanical stirrer, DFBP (18.41 g, 55.10 mmol, 1 equiv), ground to a fine powder prior to use, was dissolved in DMAc (250 mL) under an inert atmosphere. TBBT (13.80 g, 55.10 mmol, 1 equiv) was added to the solution under argon. The reaction mixture was then purged with argon and stirred at 300 rpm until complete dissolution of the monomers. Anhydrous K_2_CO_3_ (45.69 g, 330 mmol, 6 equiv) was slowly added. Temperature was raised to 100 °C. Aliquots were taken at regular time intervals and precipitated into water to check the reaction progress. After 6 h, DFBP (0.69 g, 2.75 mmol, 0.05 equiv) dissolved in DMAc (10 mL) was added via syringe transfer under argon in the reaction mixture, and the polymer solution was stirred at 100 °C for 1 h to ensure that all formed macromolecules carry nonafluorobiphenyl end groups. Thereafter, the viscous solution was precipitated into deionized (DI) water (5 L), filtered off, and rinsed several times with DI water. The polymer was then stirred in 2-propanol (500 mL) overnight followed by filtering and drying at 60 °C for 12 h and at 90 °C for 2 h under vacuum. Yield: 29.29 g, 97%.

^1^H NMR (CDCl_3_, 400 MHz, δ): 7.39 (d, 3JH-H = 2.07 Hz, 4H), 7.29 (d, 3JH-H = 1.82 Hz, 4H). 19F NMR (CDCl3, 376 MHz, δ): −141.47 (m, 4F), −136.38 (m, 4F), −140.85 (m, small peak corresponding to the polymer-end groups), −135.60 (m, small peak corresponding to the polymer-end groups).

### Synthesis of PolySAT

In a round-bottom flask (100 mL) equipped with inert gas in/outlet and a stirring bar, PolyFAT (3 g, 5.51 mmol, 1 equiv) was dissolved under argon in DMAc (60 mL) prior to the addition of SMPS (7.57 g, 48.49 mmol, 8 equiv) under argon. After complete dissolution, DBU (6.6 mL, 44.08 mmol, 8 equiv) was added to the mixture. The reaction mixture was stirred at RT for 48 h before being dialyzed in DI water, which was renewed by fresh DI water three times a day for 2 days. The purified polymer was then filtered and dried at 80 °C for 12 h and at 90 °C for 2 h under a vacuum. Yield: 3.08 g, 52%.

^1^H NMR (DMSO-*d*_6_, 400 MHz, δ): 7.39 (d, 3JH-H = 2.07 Hz, 4H), 7.34 (d, 3JH-H = 1.82 Hz, 4H), 3.10 (broad peak), 2.39 (broad peak), 1.77 (broad peak). ^19^F NMR (DMSO-*d*_6_, 376 MHz, δ): −105.05, −104.11, −99.32, −98.09, −96.85.

### Membrane Preparation of PolySAT Blended with PBI-OO

In a closed vial (100 mL) equipped with a magnetic stirrer were added PolySAT (1.5 g) and DMSO (40 mL). The mixture was left under stirring until complete dissolution of the polymer before adding PBI-OO (0.25 g) and stirring for 12 h. The solution was poured on a Teflon squared mold (*l* × *w* × *h* = 12 cm × 12 cm × 1 cm) and dried at 80 °C for 12 h and at 90 °C for 2 h under vacuum. A few drops of a 5% (w/w) HCl solution were spread onto the dried membrane film to peel it from the Teflon substrate. The wet membrane thickness was adjusted to be in the range 60–80 μm by tuning the polymer solution content (PolySAT and PBI-OO), and the measured thickness was 73 μm.

### Membrane–Electrode Assembly Preparation

The catalyst inks were prepared with a 5 wt % solid content, with a 3:1 ratio of the catalyst to Nafion ionomer in a 2-propanol:water (4:1 v/v) mixture. For the RuO2 ink, the Nafion dispersion was first added to the RuO2 powder, followed by 2-propanol:water = (4:1 v/v). For the Pt/C ink, the catalyst powder was first mixed with water to avoid combustion of the carbon particles, followed by sequentially adding the Nafion dispersion and 2-propanol. The inks were ultrasonicated for at least 10 min prior to catalyst deposition. The catalyst inks were manually spray-coated using a pneumatic airbrush (Aerotec) through a stainless steel mask with a 2 cm × 2 cm opening on the respective porous transport layers (PTLs) until the target loadings of 1 and 2 mg cm^–2^ for Pt and RuO_2_ were reached, respectively. The catalyst loadings were calculated by weighing the PTLs before and after the spray coating. The deposition temperature was set to 85 °C (for Nafion) to evaporate the solvent upon deposition.

The PSAT (P4SM-a) membrane (61 μm) was immersed in 0.5 M H_2_SO_4_ overnight to ensure full protonation of the membrane and then washed with Millipore purified water to remove the excess acid. Subsequently, the wet membrane was hot pressed between the PTLs at 80 °C and 5 MPa for 5 min.

### Proton-Exchange Membrane Water Electrolysis Cell Setup and Characterization

Water electrolysis tests were conducted in an in-house built PEM-electrolyzer cell (5 × 5 cm) using high-impact polypropylene (PP) as end plates and titanium current collectors (1 mm thick) with machined parallel flow fields (1 mm wide, channel area: 2.25 × 2.25 cm). A titanium fiber felt (2 cm × 2 cm, 0.2–0.3 mm, porosity: 53–56%, from Fuel Cell Store) and a carbon fiber nonwoven fabric (2 cm × 2 cm, 255 μm, with MPL, H23C2, from Quintech) were used as PTLs at the anode and cathode, respectively. The electrolyzer was sealed with PTFE (5 cm × 5 cm, 200 mm, from Polyfluor) and closed using a compression force of 0.8 N m. A polyimide film (Kapton 100 HN, 25 μm, from DuPont) was used between the Ti PTL and membrane to delimit the active area to 1 cm^2^. Millipore purified water (ρ > 18 MΩ cm) was circulated using a peristaltic pump (Masterflex L/S Digital Miniflex) into both anodic and cathodic compartments at 10 mL min^–1^. Independent water lines and feeding bottles were used for each compartment. The water bottles were N2-bubbled to prevent oxygen and hydrogen building up.

Galvanostatic polarization curves were recorded, and steady-state stability was tested using a two-channel Keithley 2600 SMU controlled by LabVIEW. The first channel was used to apply the current, whereas the second channel was used to measure the voltage across the PEM electrochemical cell. Electrochemical impedance spectroscopy (EIS) was performed by using a potentiostat PGSTAT30 (Autolab) equipped with a frequency analyzer (FRA) module. All measurements were taken at 60 °C. Water was circulated through the cell for 1 h to allow membrane swelling and equilibration prior to any measurements. The cell was conditioned by applying 10, 20, 50, and 100 mA cm^–2^ for 30 s and then 250 mA cm^–2^ for 30 min, followed by EIS with a frequency of 10 kHz–100 mHz at 10, 50, and 100 mA cm^–2^. Five galvanostatic polarization curves were recorded from 1 to 1500 mA cm^–2^. Each current density step was held for 2 min to allow potential stabilization, and the average of the last 10 s was taken as the potential value. The first two polarization curves were considered part of the conditioning process and are, thus, not included here. All polarization curves shown in this work represent an average of the last three polarization curves. Hydrogen crossover experiments were done while applying a current density of 250 mA cm^–2^.

The kinetic overpotential η_kin_ was calculated from the Tafel slope *b* and exchange current density *i*0 obtained by fitting the Tafel equation to *iR*-free potential up to 100 mA cm^–2^. At low current densities, the mass transfer limitations can be neglected, and thus, the kinetics overpotential can be described by the Tafel equation. The mass transfer overpotential η_mt_ was calculated according to eq 2.
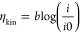
1where *i* is the current density of the cell.
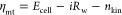
2

Gas chromatography was performed using a compact gas chromatograph CGC 4.0 (Global Analyzer Solutions-Interscience B.V.) controlled by Chromeleon 7 software (Thermo Fischer Scientific). An EL-FLOW Prestige mass flow controller (Bronkhorst Nederland) was used to control the nitrogen flow (*F*_N2_= 135 mL min^–1^) through the anode water feeding bottle where the outlet was connected to the CGC 4.0. Once every 3.8 min, a sample was injected into the gas chromatograph for analysis. A thermal conductivity detector (TCD) was used to measure the H_2_ content of the flowing gas. The gas chromatograph was calibrated at 3 points using calibration bottles with 5, 100, and 1000 ppm of hydrogen in a nitrogen balance. The Faradaic efficiency was calculated using eq 3:
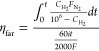
3where *C*_H2_ is the concentration of H_2_ measured with CGC 4.0 in ppm, *i* is the applied current in mA, *F* is the Faraday constant (96,485 C mol^–1^), and *t* is total time of the analysis in min.

## Results and Discussion

### Synthesis and Characterization of PolyFAT

**Scheme 1:**
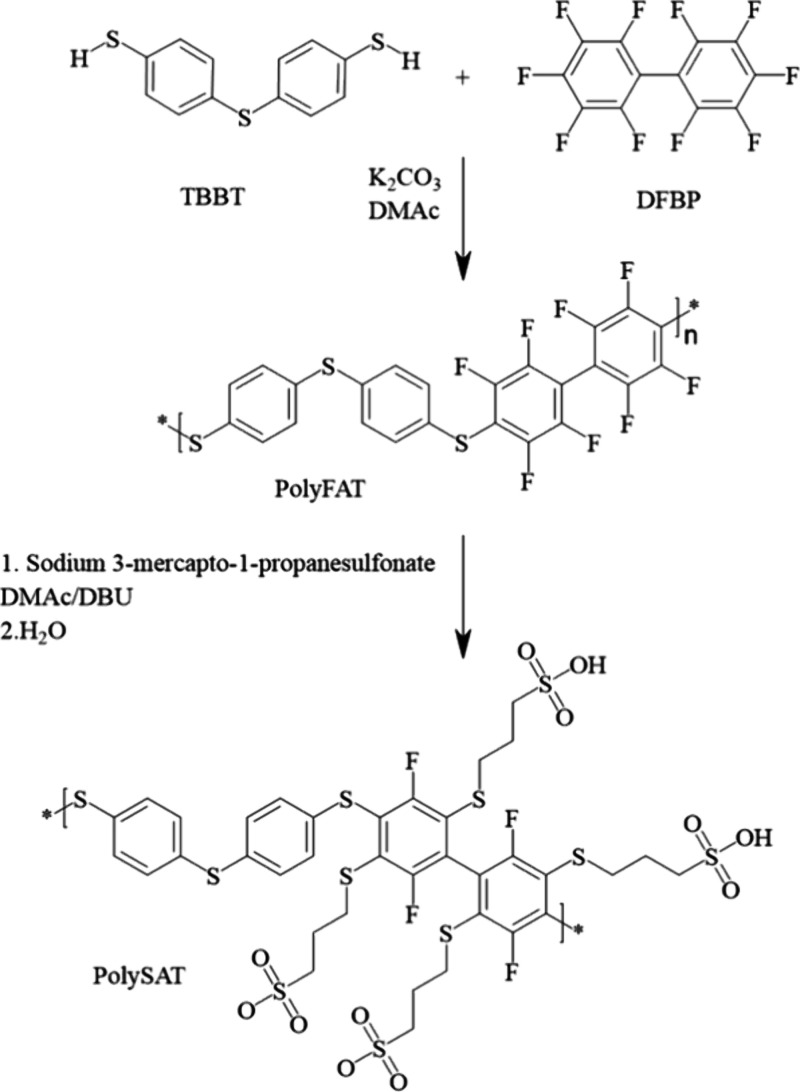
Polymerization for Obtaining PolyFAT and Sulfonation to PolySAT

In this work, we targeted the development and optimization of a simple synthetic pathway for production of sulfonated polythioethers (Scheme 1). The synthesis proceeds in two steps: (i) polycondensation of DFBP and TBBT and (ii) sulfonation of the obtained polymer.

In step (i), the [K_2_CO_3_]/[TBBT] ratio and reaction temperature were studied to determine the best experimental conditions for achieving high molecular masses by minimizing side reactions. The experimental conditions are reported in Table 1. Besides the reaction parameters, maintaining the reaction under inert atmosphere and anhydrous conditions and using an equimolar monomer ratio were found to be fundamental to achieve high molecular mass.^15^

**Table 1:** Experimental Conditions, Weight Average Molecular Mass, and Dispersity of PolyFAT Polymers within 7 h Reaction Time

#	base eq [K_2_CO_3_]/[TBBT]	temperature (°C)	*M*_w_ (Da)	dispersity (*Đ*)
PolyFAT-1	1.5	80	17,900	2.4
PolyFAT-2	3	80	53,300	3.3
PolyFAT-3	6	80	82,300	1.7
PolyFAT-4	8	80	113,000	2.6
PolyFAT-5	6	100	314,300	1.9
PolyFAT-6	6	120	321,200	16

In a first approach, the influence of the base quantity versus the amount of TBBT was investigated to identify the best conditions for the formation of thiolate reactive species enabling the polycondensation process. Potassium carbonate-to-TBBT ratio ranged from 1.5 as previously reported by Takamuku et al.^11^ to a maximum of 8. It is worth mentioning that [K_2_CO_3_]/[TBBT] = 2 is the theoretical ratio to fully deprotonate both thiol (−SH) groups of TBBT. When the reaction was performed at 80 °C, the highest molar masses were obtained with the highest ratio [K_2_CO_3_]/[TBBT] = 8.

The highest molecular weights were obtained at 100 °C. When using 6 base eq, a *M*_w_ of approximately 310 kDa was obtained keeping the dispersity controlled at 1.9 (Table 1). The high *M*_w_ was most likely due to the better solubility of both the monomers and polymer at 100 °C. At higher temperature (120 °C), the dispersity readily increased to 16 and polymerization was hard to control. The increase of dispersity might be due to side reactions such as branching or cross-linking. At higher temperature (120 °C), the dispersity readily increased to 16 and polymerization was hard to control due to a rapid increase of viscosity and even gelation in some cases. At higher temperatures, fluorine atoms in the ortho and meta positions of DFBP might also be involved in the polymerization reaction. This might lead to the formation of branches (high viscosity) and, over time, cross-linked products (gel formation).

### Synthesis and Characterization PolySAT

Sulfonation of PolyFAT to PolySAT was done by using a relatively new approach based on a well-known thiol–para fluoro “click” reaction.^16^ In this case, we used a thiol compound bearing a sodium sulfonate functional group (sodium 3-mercapto-1-propanesulfonate, SMPS). This approach has some advantages like a quick one-pot reaction, mild reaction conditions, and high sulfonation degree in comparison to sulfonation with fuming sulfuric acid^17^ or thiolation–oxidation^18^ approaches where harsh conditions may lead to polymer backbone degradation.

**Figure 1:**
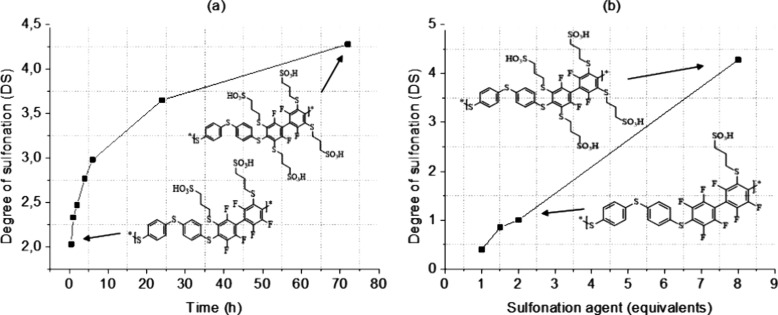
(a) Degree of sulfonation vs reaction time using 6 equiv of SMPS vs DFBP. (b) Degree of sulfonation vs equivalents of SMPS per DFBP monomer unit after 6 h reaction.

Thus, by using SMPS, sulfonation of the partially fluoroarylated PolyFAT occurred in the fluorinated polymer segments upon smooth reaction conditions and the quick kinetics of a click reaction. A study of the reaction kinetics of the functionalization (Scheme 1, second step) of PolyFAT by SMPS in DBU was carried out (Figure 1a). Samples were taken initially in short intervals and longer intervals toward the end. All samples were analyzed by ^1^H and ^19^F NMR. Integrating the ^19^F NMR peaks allowed to monitor the degree of substitution of the tetrafluorophenyl unit by sodium 3-mercapto-1-propanesulfonate side chain thioethers.

**Figure 2:**
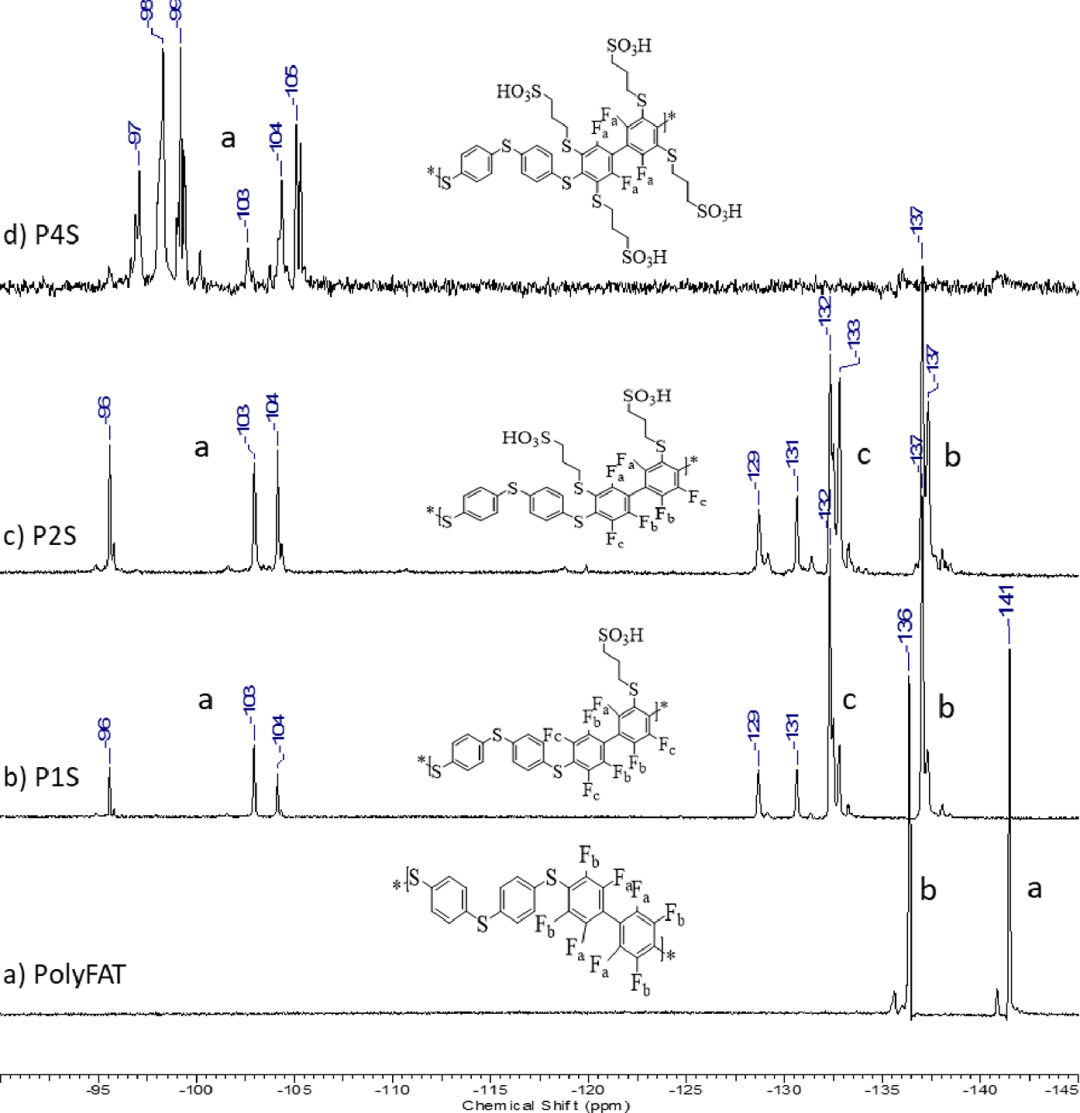
^19^F NMR of polymers: before sulfonation PolyFAT (a) and after sulfonation with one sulfonic acid P1S (b), two sulfonic acids P2S (c), and four sulfonic acids P4S (d).

The first substitution in the tetrafluorophenyl unit occurred in the first half hour (Figure 1a). Further substitution is less favorable as the electron density in the substituted phenyl ring increases due to the positive induction and mesomeric effects of the thioether group. Thus, the thiosubstituted fluorophenyl unit becomes less reactive to a further nucleophilic attack. The highest degree of substitution obtained after 72 h was 4.1 (Figure 1b), which corresponds to 2 substituted groups in every fluorinated phenyl moiety. Another study was carried out by checking the degree of sulfonation vs the equivalents of the sulfonation agent (SMPS) per DFBP unit (Figure 1b). In this case, the reaction time was fixed to 6 h at RT. The 19F NMR revealed an evolution of the peaks with an increase in the sulfonation degree (Figure 2). In the spectrum of PolyFAT (Figure 2a), the two peaks at −136 and −142 ppm correspond to the fluorine atoms in the DFBP polymer unit. With the first sulfonation after 30 min at sulfonation equiv 2 (Figure 2b), new peaks shifted downfield by 4 ppm from the original peaks and new peaks between −95 and −105 ppm were observed. As the sulfonation degree increased to 2 (Figure 2c), an intensity decrease in the initial peaks was observed. When the degree of sulfonation reached 4 (Figure 2d), no more nonsubstituted tetrafluorophenylene units were left, and therefore, no more peaks below −105 ppm were observed. On the other hand, some new peaks appeared between −95 and −105 ppm; however, an exact substitution pattern was hard to be resolved due to the statistical manner of the substitution and the large number of possible isomers.

**Figure 3:**
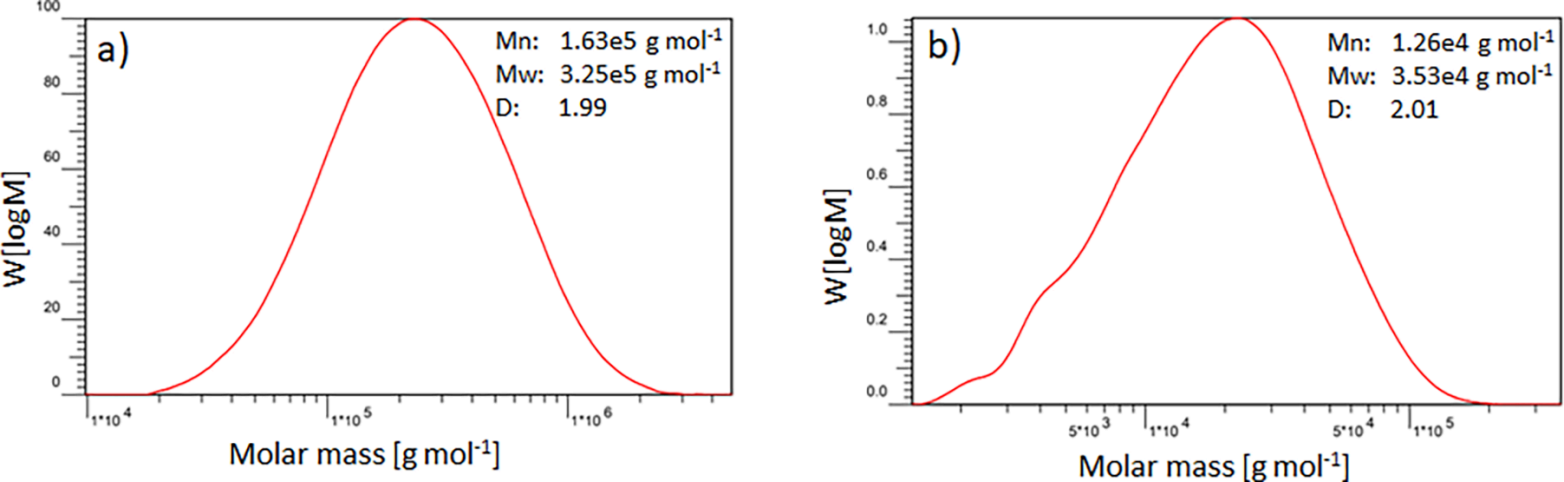
SEC molar-mass distributions of (a) PolyFAT and (b) PolySAT (P4S).

Beside functionalization degree, changes in molecular weight were monitored by recording size-exclusion chromatograms of the polymer before PolyFAT and after sulfonation with SMPS (Figure 3). A decrease of the molar masses was observed within the sulfonation step, from 320 to 25 kDa but without an increase of dispersity. This might be due to C–S bond cleavage, which is weaker than a C–C bond. Even though mild reaction conditions (RT, 20 h) are unlikely to induce backbone degradation, the high nucleophilicity of the thiolate anion might have cleaved some bonds as has been observed in similar systems.^19^ Nevertheless, the molar masses of the sulfonated polymers were still high enough to obtain stable membranes when they were blended with PBI-OO.

**Figure 4:**
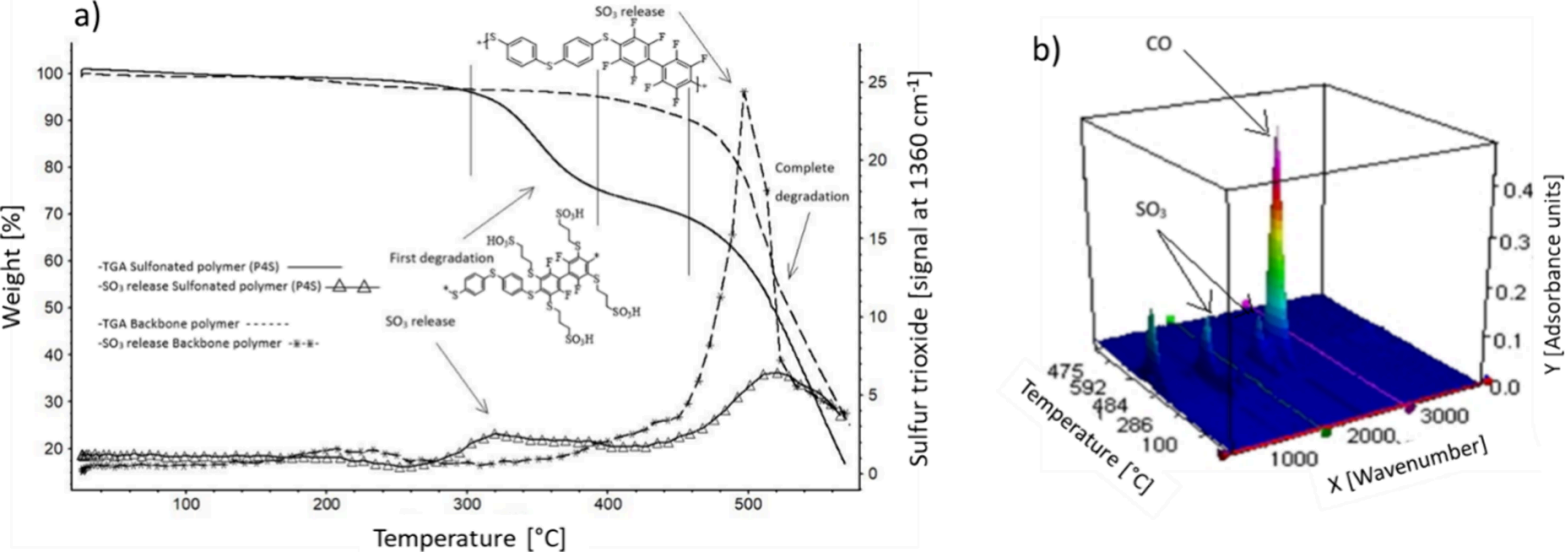
(a) TGA profiles and FTIR signal of SO_3_ gas-evolution traces for PolyFAT and PolySAT. (b) FTIR spectra recorded during TGA of PolySAT vs temperature.

Thermal stability was analyzed on both polymers before (PolyFAT) and after sulfonation (PolySAT) (Figure 4). The TGA instrument is coupled to a chamber where all exhaust gases are analyzed by FTIR. The degradation of PolyFAT started at 380 °C and progressed with a relatively low degradation rate up to 450 °C (dashed line in Figure 4a). The evolution of SO3 is initially low as inferred from the FTIR signal recorded (dashed line with stars). Above 450 °C, PolyFAT degrades more rapidly, and the FTIR signal of SO_3_ increased sharply due to thiol-phenol backbone degradation. In the case of PolySAT, the TGA profile showed two steps of degradation starting at 280 and 450 °C. There is a weight loss of approximately 30 wt % in the first degradation step that can be attributed to the desulfonation of the polymer. The four sulfonic acid groups have a combined molecular weight of 323.8 g mol^–1^, corresponding to about one-third of the molecular weight of the monomer unit (1089.3 g mol^–1^). This is identical with the weight loss observed in the first degradation step (280–400 °C). To confirm this, the FTIR spectra were examined and characteristic SO_3_ peaks at 1065 and 1390 cm^–1^ were registered. The SO_3_ FTIR profile also showed a slight increase of intensity in the FTIR spectra at 1390 cm^–1^ in the temperature region 280–400 °C (Figure 4) confirming the desulfonation. The second degradation step in the PolySAT TGA profile is ascribed to degradation of the polymer backbone.

FTIR spectra-scans plotted vs temperature can be seen in Figure 4b. Both the evolutions of the SO_3_ and CO/CO_2_ peaks were found at 1390, 2300, and 2100 cm^–1^, respectively.

### Membrane Preparation and Characterization

**Figure 5:**
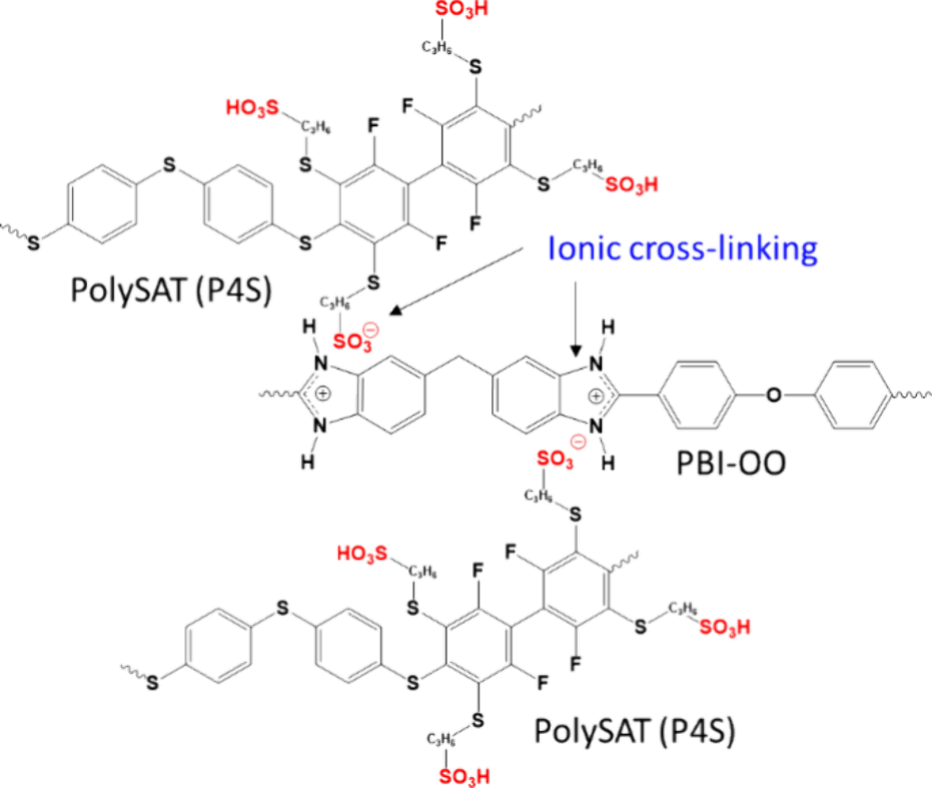
Schematic representation of ionic cross-linking of PolySAT (P4S) with PBI-OO.

Polymer electrolyte membranes based on PolySATs with various sulfonation degree were prepared by blending the PolySAT with PBI-OO. The stabilization of the membrane is due to ionic cross-linking between the polymer acid PolySAT and polymer base PBI-OO caused by a proton transfer from the acid to the base and formation of an ion pair (Figure 5).

The method used for membrane preparation was doctor blading of the polymer solution on a glass plate.^20^ The P1SM membrane was based on P1S (i.e., PolySAT with a 1 sulfonate side chain) with IEC = 1.47 mequiv g^–1^ resulted in a stable polymer film (Table 2). In the case of PolySAT with 2 end-sulfonated side chains (P2S, IEC = 2.45 mequiv g^–1^), the polymer was water-soluble but brittle in the pure form and had therefore to be blended with PBI-OO to obtain excellent film-forming properties (P2SM membrane). Blending of P2S with PBI-OO (P2S:PBI-OO = 93:7 wt %) decreased the IEC from 2.45 to 1.96 mequiv g^–1^. Blending of P4S with PBI-OO resulted in stable membranes (P4SM) when the PBI-OO content was above 15 wt %. In this case, the IEC dropped down to 1.09 mequiv g^–1^ resulting in a very stable blend membrane. It is worth mentioning that there was a little discrepancy between the calculated and titrated IEC values especially for the membranes with larger PBI-OO contents (P4SM-a, -b, and -c). This might be attributed to the encapsulation effect of PBI-OO, which will restrict the fast ion-exchange in some membrane segments.

**Table 2:** Characteristics of Homopolymer and Blend Membranes

polymer	membrane	PBI-OO(wt %)	IEC_Calc._(meq g^–1^)	IEC_Titr._(meq g^–1^)	conductivity (mS cm^–1^)	water uptake (wt %)
P1S	P1SM	0	1.48	1.57	135	*a*
P2S	P2SM	7	2.00	1.96	202	*a*
P4S	P4SM-a	15	2.50	1.09	179	57
P4S	P4SM-b	19	2.17	1.12	136	42
P4S	P4SM-c	22	1.90	1.13	64	30

The water uptake (WU) was recorded for all the membranes; however, P1SM and P2SM became too strongly swollen and the swollen membranes were too soft for an accurate WU determination. For the P4SM membranes, the WU was in the range 60–30 wt % and was decreasing with the increase of the PBI-OO content in the membrane.

Ionic conductivity was determined by EIS in 0.1 M sulfuric acid at RT. For P1SM and P2SM, the conductivity was found to be very high, which can be explained by the high IEC and WU. For comparison, the conductivity of Nafion NRE-212 was recorded to be 128 mS cm^–1^ at the same conditions. In the case of P4SM membranes, conductivity was varying from 180 to 60 mS cm^–1^ decreasing with the increase of the PBI-OO content, which can be explained as the higher the PBI-OO the higher the hindrance of some sulfonic groups having also a decreasing effect on the WU (Table 2). Thus, the highest conductivity for a membrane was recorded for P4SM-a (179 mS cm^–1^).

**Figure 6:**
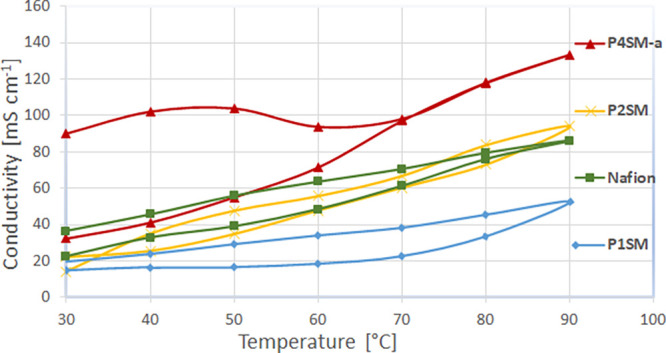
Membrane proton conductivity with temperature at 90% RH.

To determine the impact of the temperature on the conductivity, EIS in an atmosphere with constant relative humidity (RH = 90%) was recorded in a conductivity test station (Figure 6). It is worth noting that the conductivities in Table 2 are higher than the data plotted in Figure 6. This is due to the differences in the measurement conditions where the membranes were conditioned and measured in 0.1 M H_2_SO_4_ at RT (data presented in Table 2) versus a humidified atmosphere (data in Figure 6). Polymer membranes P1SM, P2SM, and P4SM-a were tested and compared to Nafion NRE-212. The proton conductivity increased with the temperature and ranged between 14 and 90 mS cm^–1^ at 30 °C and 52–133 mS cm^–1^ at 90 °C. The conductivity of Nafion NRE-212 is very similar to that of P2SM with IEC = 1.96 mequiv g^–1^. The highest conductivity was obtained for P4SM-a reaching 133 mS cm^–1^ at 90 °C. In all cases, hysteresis was observed when cycling the temperature from 30 to 90 °C and then back to 30 °C. This might be due to some irreversible swelling/deswelling features of the membranes.

**Figure 7:**
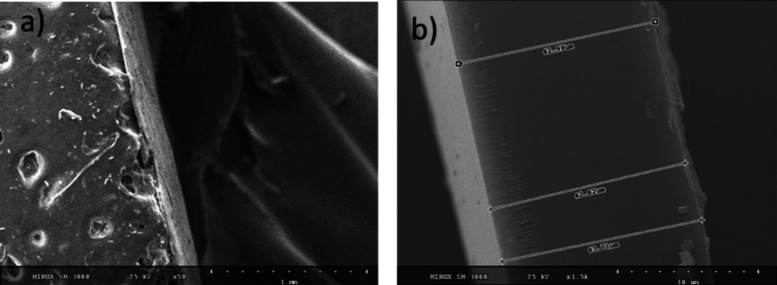
Membrane P4SM SEM x50 (a) and P4SM-a SEM x1.5k (b).

To check for surface and morphology changes or structuring, the membrane’s surfaces and cross sections were examined by scanning electron microscopy (SEM) (Figure 7). A smoother side was observed toward the Teflon substrate, onto which the membrane was cast (Figure 7a). Small irregularities were observed on both sides and may be attributed to dust or small surface defects. Nevertheless, no structuring or morphology patterns were observed on either side of the membranes. The SEM images recorded on the cross-section confirmed the formation of a dense polymer film with a thickness of about 43 μm (thickness of membrane in ambient conditions was 61 μm). The cross-section images did not reveal any structuring or morphology anisotropy.

### Proton-Exchange Membrane Water Electrolysis Test (PEMWE)

**Figure 8:**
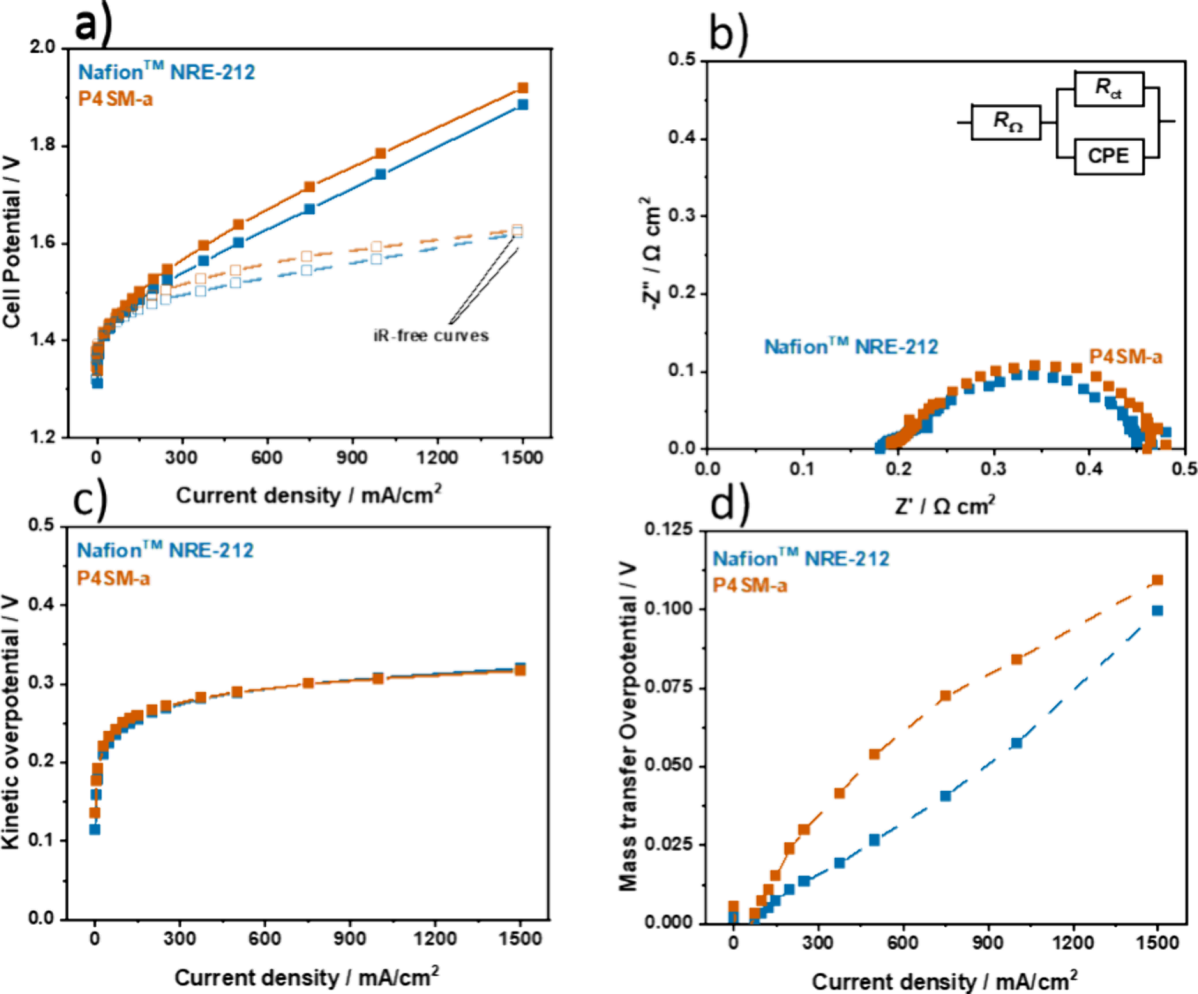
Electrochemical characterization of P4SM-a and Nafion NRE-212 carried out at 60 °C. (a) Polarization curves and *iR*-free polarization curves. (b) Electrochemical impedance spectroscopy at 100 mA cm^–2^. Inset: the equivalent electric circuit used to fit the EIS data. (c) Kinetic overpotential. (d) Mass transfer overpotential.

The P4SM-a membrane was further tested in a PEMWE cell and compared with Nafion NRE-212. This membrane was chosen, as it showed the best proton conductivity and film-forming properties among all prepared membranes. As catalysts, we used RuO_2_ for the oxygen evolution reaction and Pt/C for the hydrogen evolution reaction. RuO_2_ has higher activity but a lower long-term stability in comparison to that of IrO2. The membrane–electrode assemblies were prepared using a catalyst-coated substrate (CCS) approach, meaning that the catalyst inks were spray-coated directly on the PTLs, followed by hot-pressing the PTLs against the membranes. Figure 8a shows the polarization curves recorded for both membranes at about 60 °C. The P4SM-a membrane reached a cell potential of 1.784 V at 1 A cm^–2^ and, in general, achieved higher potentials at every probed current density than Nafion.

The membranes were further characterized by EIS (Figure 8b) at 100 mA cm^–2^. The EIS spectra were fitted to the equivalent electrical circuit shown in the inset of Figure 8b, where *R*_Ω_ is the ohmic resistance, *R*_ct_ is the charge transfer resistance, and CPE is a constant phase element. *R*_Ω_ comprises the electronic resistance of the electrodes and cell hardware and the membrane ionic resistance, and it is the high-frequency intercept on the real axis of the Nyquist plot. The charge transfer resistance *R*_ct_ consists primarily of kinetic characteristics at low current densities (such as 100 mA cm^–2^) and provides information about the impact of the membrane–catalyst layer interface. The *R*_ct_ can be obtained from the low-frequency intercept on the real axis in the Nyquist plot and subtracting *R*_Ω_.^21^ The experiment revealed that *R*_Ω_ was about 0.190 Ω cm^2^ for the Nafion membrane and 0.199 Ω cm^2^ for P4SM-a. The membrane resistance is usually the main contributor to the ohmic resistance because the electrodes and cell hardware are much more conductive. The membrane resistance is given by *R*_m_ = *d*/σ_m_, where σ_m_ is the ionic conductivity and *d* is the membrane thickness. Based on the higher σ_m_ for P4SM-a than for Nafion (Figure 8), the measured value of *R*_Ω_ for P4SM-a was expected to be lower. The membranes will further swell inside the electrochemical cell due to water uptake, resulting in an increase in membrane thickness. The WU at 60 °C of Nafion was 34.4%, whereas P4SM-a had a WU of 105%, leading to a larger thickness increase and slightly higher *R*_Ω_.^22,23^

In contrast, the *R*_ct_ was only marginally lower for the P4SM-a (0.280 Ω cm^2^) than for Nafion (0.286 Ω cm^2^) suggesting that the catalyst layer–membrane interface slightly improved, which can be attributed to the higher IEC of P4SM-a. Also, the higher water uptake and swelling contributed to lower *R*_ct_ values as it leads to larger compression between the membrane surface and catalyst-coated porous transport layers (PTL). The similar *R*_ct_ suggests identical activation overpotential at low currents, which is consistent with the kinetic overpotential calculated from the *iR*-free polarization curves (see Experimental Section) (Figure 8c). The mass transfer overpotential η_mt_ was calculated by subtracting the kinetic overpotential from the *iR*-free potential (Figure 8d). Similarly to the ohmic overpotential, η_mt_ was also higher for PS4M-a. Here, the excessive swelling of this membrane might have caused the membrane to infiltrate the pores of PTLs, resulting in less space for gas escape and thus increased the mass transfer overpotential.

**Figure 9:**
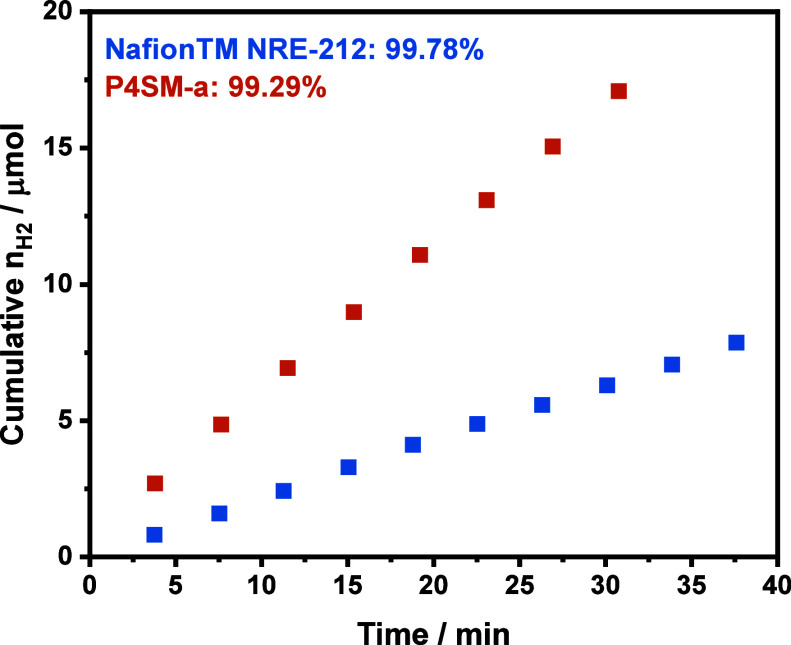
Cumulative hydrogen amount detected in the anode compartment of the water electrolysis setup by in-line gas chromatography during continuous operation at 250 mA cm^–2^. The respective Faradaic efficiencies obtained for the membranes are shown in the legend.

A good PEM provides not only excellent proton conductivity but also safe separation of the evolved gases: hydrogen and oxygen, i.e., they should have low gas permeabilities. A low gas crossover will avoid two main issues: an explosive gaseous mixture (H_2_/O_2_) and a decrease of the water electrolysis Faradaic efficiency η. Therefore, the anode compartment was connected to a gas chromatograph for hydrogen detection during continuous operation at a current density of 250 mA cm^–2^ also at 60 °C. The cumulative hydrogen amount found in the anode compartment and the respective Faradaic efficiencies are shown in Figure 9. The P4SM-a membrane showed a larger hydrogen crossover that negatively impacted the Faradaic efficiency by roughly 0.5% and resulted in a concentration of 1.4 mol H_2_% in the evolved oxygen stream. This loss in Faradaic efficiency may seem small; however, for large scale operation (several kA), it can represent large losses of hydrogen. A higher membrane thickness would ensure less gas crossover, however, that would also result in an undesirable increase in the ohmic resistance.

The hydrogen permeability of each membrane was calculated as reported by Schalenbach et al. to further understand the gas crossover, independent of the membrane thickness. It was found that the hydrogen permeability for Nafion is 0.396 × 10^–10^ mol cm^–1^ s^–1^ bar^–1^, which is consistent with typical values found in the literature.^24^ The permeability of P4SM-a was 1.01 × 10^–10^ mol cm^–1^ s^–1^ bar^–1^, almost three times higher than that for Nafion, mostly ascribed to higher water uptake of this membrane as well. Schalenbach et al. suggested that gas permeation occurs via a mixed pathway in wet membranes, i.e., both through the solid ionomer phase and through the water-filled pores. Since water has higher gas permeability than the solid ionomer phase, high water uptake results in larger pores, ultimately increasing overall permeation through the membrane. This emphasizes the importance of water uptake control in membrane ionomers while still achieving high ion transport properties to avoid dangerous mixtures and lower Faradaic efficiencies.

## Conclusions

In this study, new polythioethers were designed to obtain highly conducting and stable PEMs for water electrolysis application. In a first step, a polycondensation in mild conditions was optimized to result in high-molecular-mass (∼300 kDa) poly(arylene thioethers) incorporating a perfluorinated biphenyl unit in the main chain. In a second step, sulfonation via the click reaction at room temperature between a sulfonic acid bearing thiol and the perfluorinated biphenyl unit in the polymer chain was used to obtain a class of polymers with multiple sulfonic acid side chains. At a high relative humidity of 90%, PEMs made using these polythioethers showed good proton conductivity for IECs ranging from 1.57 to 1.96 mequiv g^–1^ and reached 90 and 133 mS cm^–1^ at 30 and 90 °C, respectively. This conductivity is higher than Nafion, being 36 and 86 mS cm^–1^ under the same conditions. Additionally, their performance in a water electrolysis experiment was quite close to that of Nafion NRE-212 (50 μm dry thickness) despite the higher thickness of the experimental membranes, because due to the high swelling of the polythioether membranes, higher potentials and hydrogen crossover were obtained. This research provides a promising stand for further non-PFSA proton-conducting polymers, especially emphasizing the need to design ionomers and, thus, membranes that have great ionic transport properties but also have low water uptake and gas permeabilities while fully hydrated and under operation.
